# Dosimetric comparison of hybrid IMRT and different arc techniques VMAT after breast‐conserving surgery for left‐sided breast cancer

**DOI:** 10.1002/acm2.70257

**Published:** 2025-08-29

**Authors:** Xin Chen, Wei Li, Shi‐Long Song, Nan‐Nan Qin, Xian‐Xiang Wu, Han‐Fei Cai

**Affiliations:** ^1^ Department of Radiation Oncology the First Affiliated Hospital of Bengbu Medical University Bengbu Anhui China

**Keywords:** breast cancer, dosimetry, hybrid intensity‐modulated radiation therapy, volume‐modulated arc therapy

## Abstract

**Purpose:**

This study aimed to comprehensively compare the dosimetric characteristics of three different radiotherapy techniques—hybrid intensity‐modulated radiotherapy (hy‐IMRT), tangential volumetric‐modulated arc therapy (t‐VMAT), and continuous volumetric‐modulated arc therapy (c‐VMAT)—used after breast‐conserving surgery for left‐sided breast cancer in the target area and organs at risk (OARs) after breast‐conserving surgery for left‐sided breast cancer. This evaluation aims to provide a solid basis for individualized radiotherapy planning in clinical practice.

**Methods:**

Twenty female patients who underwent breast—conserving surgery for left—sided breast cancer were retrospectively selected. These patients were treated with hy‐IMRT, t‐VMAT, or c‐VMAT. The doses received by the target area and OARs were precisely evaluated. Additionally, the dose distribution in normal tissues and treatment time of the three radiotherapy plans were carefully compared.

**Results:**

All three techniques could meet the target zone dose requirements. In the planning gross tumor volume (PGTV), the average dose of hy‐IMRT was the highest. c‐VMAT demonstrated the best homogeneity index (HI) and conformity index (CI). For the planning target volume (PTV), c‐VMAT also showed outstanding performance in terms of homogeneity and conformity (*p* < 0.001). Regarding OAR doses, although there was no significant difference in the mean cardiac dose among the three techniques (*p* = 0.69), c‐VMAT had lower values in cardiac V10 dose volume, mean coronary left anterior descending (LAD) artery dose, and V20, V30, and V40 dose volumes, irradiating less of the cardiac low—dose and high—dose regions. c‐VMAT had lower left lung V30 and V40 doses, but its mean dose to the right lung was significantly higher than that of the other two groups. In terms of treatment time, t‐VMAT was significantly shorter than the other two groups, indicating the highest efficiency (*p* < 0.001).

**Conclusion:**

C‐VMAT exhibits obvious advantages in target area uniformity, conformality, and protection of the heart and the affected lung. However, its significant dose impact on the right lung cannot be ignored and requires further attention. On the other hand, t‐VMAT has a remarkable advantage in treatment time. This study offers valuable references for clinicians to select the most suitable radiotherapy technique according to patients' specific conditions, such as tumor location, size, and OAR anatomical structures, aiming to optimize treatment outcomes and minimize adverse effects.

## 1 INTRODUCTION

Breast cancer, as one of the most prevalent malignant tumors among women, poses a significant threat that cannot be overlooked. It not only affects patients’ physical health but also profoundly impacts their overall quality of life. According to statistics, in 2022, there were 2.3 million new cases of breast cancer globally, accounting for 25% of new cancer cases in women; among these, there were 670,000 deaths from breast cancer, representing 15.5% of cancer deaths in women.^1^ In China, there were approximately 357,200 new cases of breast cancer in 2022, ranking second in the incidence of malignant tumors among women. Additionally, more than 30,000 young breast cancer patients aged 15–39 are newly diagnosed each year, with the incidence rate showing a gradual upward trend.^2^ In the diversified treatment strategies for breast cancer, radiotherapy serves as a core component, making significant contributions to controlling the probability of recurrence in the lesion area and improving patient prognosis. Through precise irradiation with high‐energy rays, radiotherapy can effectively inhibit the proliferation of tumor cells. Studies have shown that the rational application of radiotherapy not only enhances disease control efficacy but also significantly prolongs patients’ survival cycles. With the increasing demand for breast aesthetics and functionality among women, breast‐conserving surgery (a procedure that removes the tumor while preserving the breast) has become increasingly prominent postoperative whole‐breast radiotherapy is an essential step, effectively reducing metastasis and local recurrence rates.^3^, ^4^ Intensity modulated radiation therapy (IMRT) and volumetric modulated arc therapy (VMAT) are the most widely used treatment modalities for breast cancer radiotherapy.^5^, ^6^ Among them, patients who have undergone breast‐conserving surgery often receive hybrid IMRT (hy‐IMRT) due to the larger volume of retained breast glandular tissue, which is susceptible to respiratory motion. This approach effectively reduces the risk of missing the target while improving the conformity and uniformity of the target area. VMAT technology, known for its short treatment time and excellent dose distribution, has been widely applied in clinical practice. In breast‐conserving radiotherapy, VMAT techniques can be divided into two main categories based on their arc delivery methods: continuous VMAT (c‐VMAT) and tangent VMAT (t‐VMAT).^7^ The c‐VMAT technique employs a continuous arc irradiation method, maintaining beam continuity throughout the entire treatment process, while the t‐VMAT technique utilizes a segmented tangential arc irradiation method, dividing the entire treatment process into multiple independent arc segments. This classification method based on arc modes not only aids in more accurately describing the characteristics of VMAT technology but also provides a theoretical foundation for subsequent technical optimization and clinical applications.^8^ Currently, there is limited research on these three techniques in the context of post‐breast‐conserving surgery for left breast cancer. This study aims to explore the dose distribution in target areas and organs at risk using these three techniques after breast‐conserving surgery for left breast cancer, offering a more optimal individualized radiotherapy plan for clinical practice.

## 2 METHODS

### 2.1 Patient selection

A retrospective collection of 20 female patients, aged 23–70 years, with a median age of 48.5 years, after breast‐conserving surgery for left‐sided breast cancer who were treated in our oncology radiotherapy department from January 2023 to December 2024 was performed. Inclusion criteria: (1) the patients’ tumor primary foci were located in the left breast, and the postoperative pathological type was infiltrative; (2) the clinical staging was based on the UICC/AJCC 9th edition of breast cancer staging as T1‐2N0M0^9^, ^10^; (3) the patients’ arms could be freely lifted up, and the posing repetitively was good. All patients were treated for the first time and signed an informed consent for radiotherapy.

### 2.2 Immobilization and simulation

The patient's position was fixed in the supine position with arms abducted and raised above the head. A thermoplastic film and styrofoam were used for individualized immobilization. After marking, a 4D‐CT scan was performed with 5 mm slice thickness. and the acquired image data were transferred to the Pinnacle^3^9.8 treatment planning system (Philips, The Netherlands) via the network, and the equipment used was the Elekta Infinity linear accelerator (Elekta, Sweden).

### 2.3 Delineation of target area and organs at risk

**FIGURE 1:**
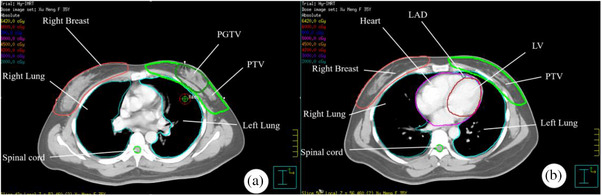
Example of target area and organs at risk outlined in a case of a patient. (a) Cross section of target area, (b) cross section of heart.

The scope of target area was outlined in the treatment planning system by doctors at or above the deputy high‐level of the radiotherapy department of our hospital, and the target area was outlined with strict reference to the guidelines of the National Cancer Center in conjunction with the patient's imaging data.^11^, ^12^ The clinical target volume (CTV) included the entire breast glandular tissue, the upper boundary did not exceed the sternoclavicular joint, the lower boundary extended to the disappearance of the breast elevation, the anterior boundary was 0.5 cm from the subcutaneous area, and the posterior boundary was located in the anterior part of the pectoralis major muscle fascia, but it did not include the rib cage and intercostal muscle structures. The PTV was created by expanding the CTV uniformly by 0.5 cm in all directions, with the anterior boundary retracted to 0.3 cm beneath the skin surface, with the external boundary not exceeding the mid‐axillary line and the internal boundary not exceeding the thoracic‐costal joints, and was treated with a dosage regimen of 50 Gy/25 fractions. The tumor bed location was determined by an intraoperative silver‐plated CTV. The location of the tumor bed was localized by intraoperative silver clip marking and postoperative hematoma area. The clinical target volume tumor bed (CTV‐TB) was established by using the tumor bed as the reference and placing 1.0 cm outward in all directions without exceeding the CTV. Subsequently, the CTV‐TB was used as the basis for uniformly expanding 0.5 cm in all directions, and the anterior boundary was also adjusted to 0.3 cm subcutaneously, thus generating the planning gross tumor volume (PGTV), which was irradiated using a dose division scheme of 60 Gy/25fractions. Organs at Risk (OAR) were generated uniformly by the automatic outlining software, and then reviewed and modified by the supervising physician, including the whole heart, left ventricle (LV, referring to the ventricular wall, not the blood chamber content)left anterior descending coronary artery (LAD), both lungs, the right breast, and the spinal cord, as shown in Figure 1.

### 2.4 Plan design

**FIGURE 2:**
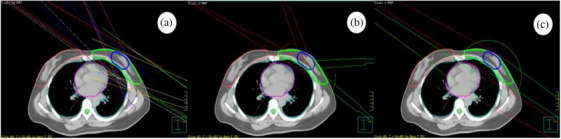
Three groups of planned irradiation field directions; (a) hy‐IMRT, (b) t‐VMAT, (c) c‐VMAT.

In the Pinnacle^3^9.8 treatment planning system, all plans used 6 MV X‐ray with synchronous dosing, and the dose rate was 600 MU/min. For hy‐IMRT, the tangent fields were designed according to the shape of the target area. The tangent angle of the conformal field was standardized to meet the incident dose requirements of the target area while minimizing the irradiated volume of the affected side's lungs and the heart. Then, the PTV was conformed along the direction of the tangent field to reduce the impact of respiratory movement. Finally, to further minimize the effect of respiratory movement and prevent off‐target, the conforming field was expanded 20 mm to the lateral chest wall. The static intensity adjustment field is formed by retracting the two fields on each side of the main field by 10–15° to create two intensity adjustment fields, and at the same time increase the intensity adjustment field of the fixed lead door specifically for the PGTV to form a 5‐field hybrid static intensity adjustment plan. In this plan, the prescription doses for the conformal field, the intensity‐tuning field, and the fixed‐lead‐gate intensity‐tuning field are 160, 40, and 40 Gy, respectively. For t‐VMAT, the two conformal tangent fields are taken as the starting and termination angles. The plan design avoids intermediate arcs that penetrate the lungs for direct irradiation, and two segmental arcs with a range of 30°–40°, which is similar to the “scissors shape,” are formed. This technique can be used and fine‐tuned according to the shape of the target area. c‐VMAT: continuous double arc, also using the two conformal tangential fields as the start and end angles, with the first section clockwise and the second section counterclockwise. This configuration is shown in Figure 2. In practical clinical applications, the successive arcs will directly irradiate the lungs, heart and healthy mammary glands, all the plans will have volume constraints in the low‐dose region at the lungs, heart and healthy mammary glands to protect the important critical organs.^13^ The dose constraints and optimization parameters were the same for all three plans, requiring the prescribed dose to reach at least 95% of the target area volume. The OAR dose limits were defined as follows: left lung V20 ≤ 30%, V30 ≤ 20%, V5 ≤ 50%; heart V30 ≤ 10%; LV V6 ≤ 30%; LAD V40 ≤ 50%.

### 2.5 Plan evaluation

The dose‐volume histogram (DVH) was used to evaluate the dose received by the target area and organs at risk. The target area parameters include the approximate maximum dose (D2), mean dose (Dmean), homogeneity index (HI), and conformity index (CI). The calculations for HI and CI are provided by Formulas (1) and (2), respectively.

(1) $$\mathrm{HI}=\frac{D_{2}\%-D_{98}\%}{D_{50}\%}\times 100\%$$

D_2%_, D_98%_, and D_50%_ represent the doses received by 2%, 98%, and 50% of the target volume, respectively. The homogeneity index (HI) is calculated based on these values to reflect the dose uniformity within the target. The closer the HI is to 0, the better the target volume homogeneity.^14^

(2)

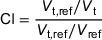




*V*_t_ is the volume of the target area, *V*_t,ref_ is the volume of the target covered by the isodose line, and *V*_ref_ is the volume of all volumes spared by the isodose line, with a CI closer to 1 representing a better conformality of the target area.^6^

The dose assessment of the organs at risk covers several key indicators: the mean dose to the left lung, heart, left ventricle (LV), and left anterior descending branch (LAD) (Dmean), and the irradiated volumes V5, V10, V20, V30, and V40; the right lung Dmean, V5, and V10; the spinal cord Dmax; and the right breast Dmean.

### 2.6 Statistical analysis

SPSS26.0 statistical software was used to process the data of the three groups of samples, firstly verifying their normality characteristics, and then implementing one‐way ANOVA (One‐way ANOVA). The data results were presented as mean ± standard deviation (mean ± SD), and the least significant difference (LSD) method was utilized for multiple comparisons between the groups, with *p* < 0.05 as the difference was statistically significant.

## 3 RESULTS

**FIGURE 3:**
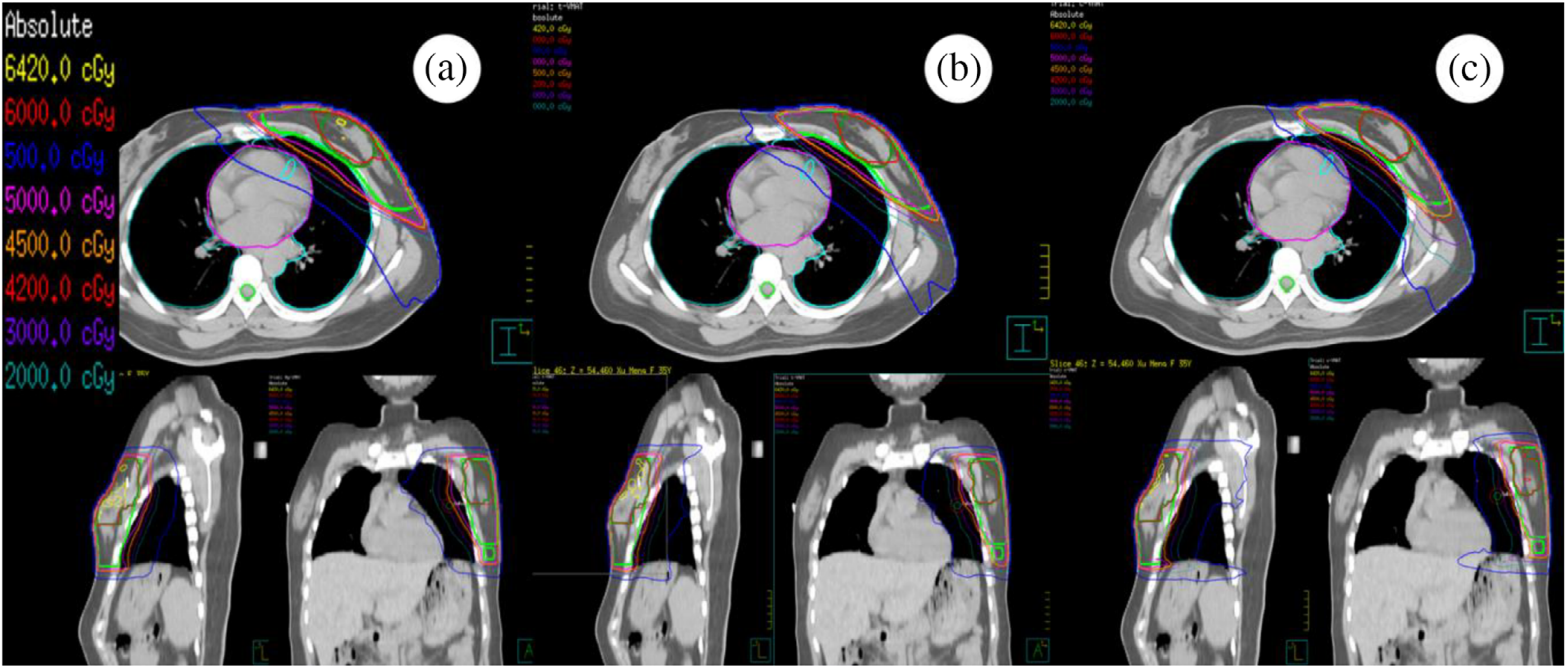
Planned dose distribution for the three groups of patients; (a) hy‐IMRT, (b) t‐VMAT, (c) c‐VMAT.

**FIGURE 4:**
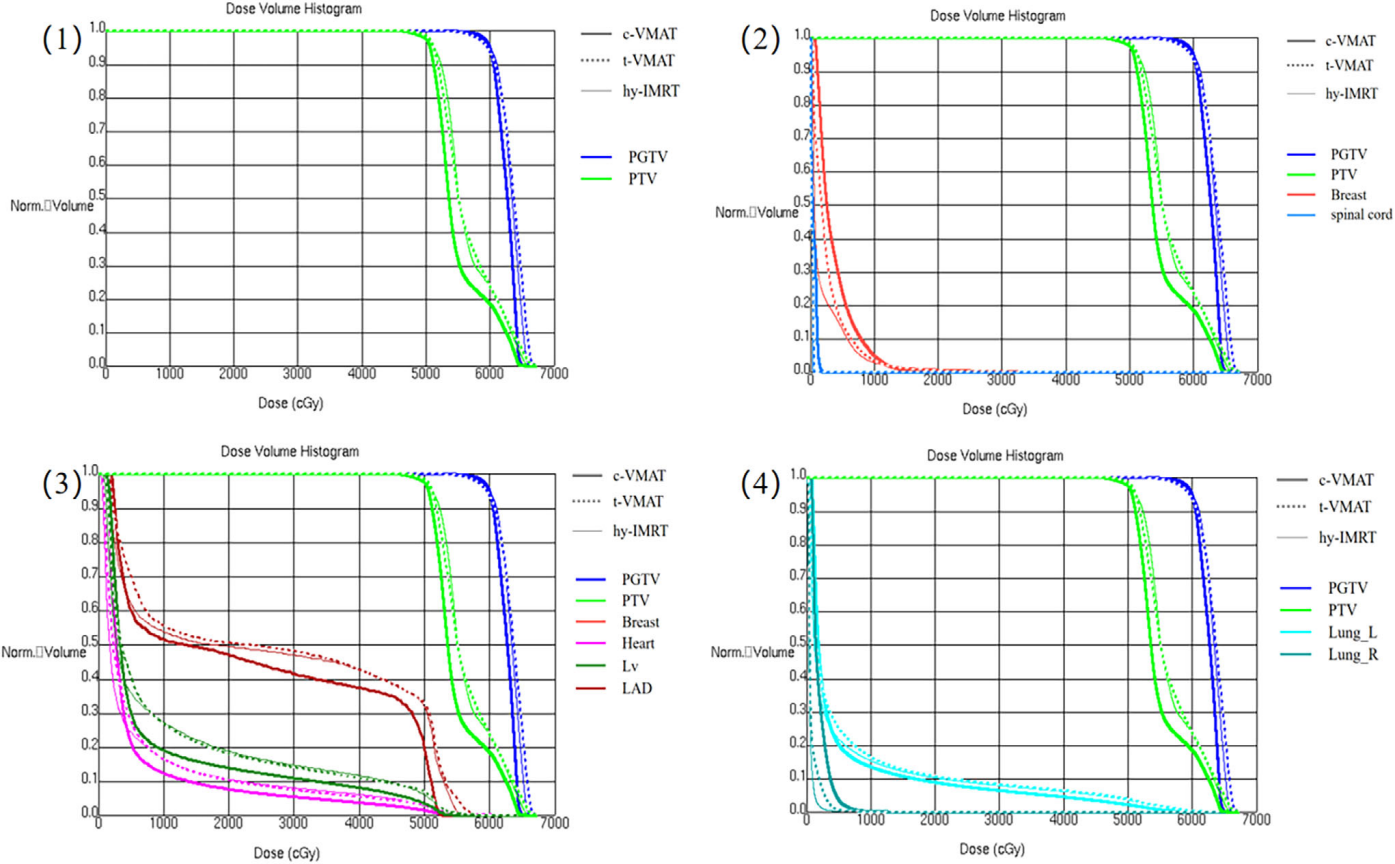
DVH maps of the three groups of the patient's program; (1): target area (2): breast and spinal cord (3): heart, LAD, LV (4) Lung_L, Lung_R.

**FIGURE 5:**
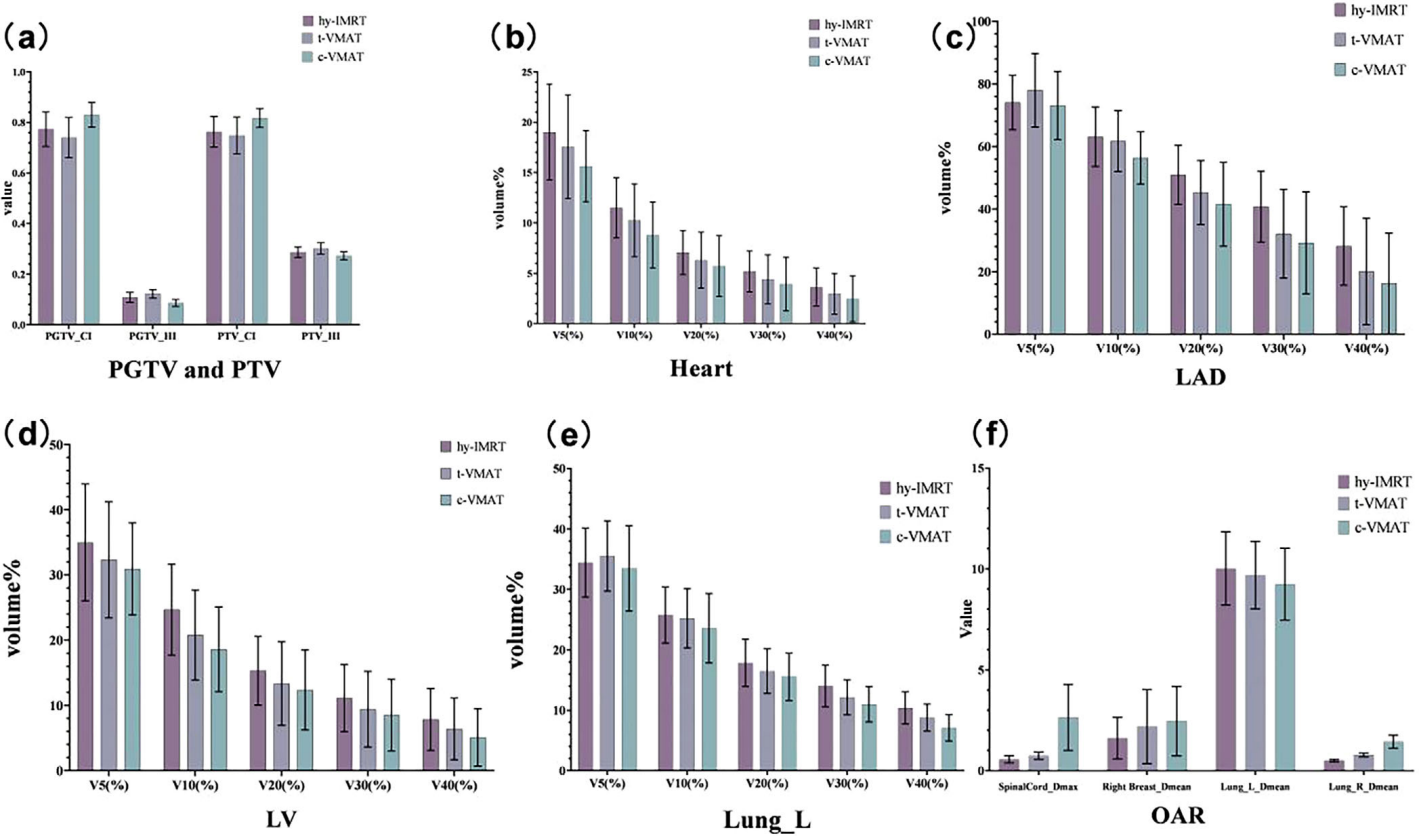
The three groups were planned to be significantly different after one‐way ANOVA, followed by multiple comparisons of the resulting chi‐sets. (a) PTV and PGTV target area fitness and homogeneity, (b) cardiac dose‐volume parameter, (c) LAD dose‐volume parameter, (d) LV dose‐volume parameter, (e) left lung dose‐volume parameter, (f) spinal cord, right lung, and right breast dose‐parameters.

The hy‐IMRT, t‐VMAT, and c‐VMAT plans met the dose requirements for the target area and OAR. The dose distributions of target area and OARs are shown in Figure 3, the DVH distribution curves are shown in Figure 4, and the parameters and the significance of differences are shown in Figure 5.

### 3.1 PTV coverage

The detailed dosimetric parameters for the target volumes are summarized in Table 1. For the planning gross tumor volume (PGTV), the hy‐IMRT plan delivered the highest mean dose (62.66 ± 0.47 Gy), which was significantly greater than that of both t‐VMAT (62.11 ± 0.68 Gy) and c‐VMAT (61.92 ± 0.43 Gy) (*p* < 0.001). Regarding dose homogeneity, c‐VMAT showed the most favorable performance, with the lowest homogeneity index (HI = 0.086 ± 0.014), while t‐VMAT had the highest HI (0.122 ± 0.017) (*p* < 0.001). In terms of conformity, c‐VMAT again achieved the best results (CI = 0.83 ± 0.049), whereas t‐VMAT demonstrated the lowest conformity index (0.74 ± 0.080), with all differences statistically significant (*p* < 0.001). For the planning target volume (PTV), hy‐IMRT and t‐VMAT yielded comparable mean doses (56.12 ± 0.69 Gy and 56.14 ± 0.65 Gy, respectively), while c‐VMAT delivered a significantly lower mean dose (55.30 ± 0.53 Gy; *p* < 0.001). In terms of homogeneity, c‐VMAT again exhibited superior performance (HI = 0.273 ± 0.016), and t‐VMAT showed the least favorable result (HI = 0.302 ± 0.023; *p* < 0.001). For conformity, c‐VMAT had the highest CI (0.817 ± 0.037), and t‐VMAT the lowest (0.748 ± 0.073), with all differences being statistically significant (*p* < 0.001). Further pairwise comparisons revealed no significant differences between hy‐IMRT and t‐VMAT in terms of PGTV conformity index or PTV mean dose, homogeneity, and conformity (all *p* > 0.005).

**TABLE 1:** Dosimetric comparison of target areas in three plans (mean ± SD).

Parameters	hy‐IMRT ($\bar{x}$ ± *s*)	t‐VMAT ($\bar{x}$ ± *s*)	c‐VMAT ($\bar{x}$ ± *s*)	*p* value	*P(LSD)*		
PGTV					*P*_1_	*P*_2_	*P*_3_
D_mean_ (Gy)	62.66 ± 0.47	62.11 ± 0.68	61.92 ± 0.43	<0.001	0.002	<0.001	0.272
HI	0.108 ± 0.02	0.122 ± 0.017	0.086 ± 0.014	<0.001	0.01	<0.001	<0.001
CI	0.773 ± 0.068	0.74 ± 0.080	0.83 ± 0.049	<0.001	0.12	0.01	<0.001
PTV							
D_mean_ (Gy)	56.12 ± 0.69	56.14 ± 0.65	55.3 ± 0.53	<0.001	0.92	<0.001	<0.001
HI	0.287 ± 0.021	0.302 ± 0.023	0.273 ± 0.016	<0.001	0.095	0.078	<0.001
CI	0.763 ± 0.061	0.748 ± 0.073	0.817 ± 0.037	<0.001	0.410	0.006	<0.001

*Note*: *P*_1_: hy‐IMRT vs t‐VMAT. *P*_2_: hy‐IMRT vs c‐VMAT. *P*_3_:t‐VMAT versus c‐VMAT.HI: homogeneity index; CI: conformity index; Dmean: mean dose.

### 3.2 Dose to the OARs

Table 2 details the dosimetric parameters of the heart and its at‐risk substructures for the three planning techniques. The mean heart dose showed no significant difference among the three techniques (*p* = 0.69). However, regarding the V10 dose volume, c‐VMAT (8.8 ± 3.27%) was significantly lower than hy‐IMRT (11.49 ± 2.97%), with a statistically significant difference (*p* = 0.013), indicating that c‐VMAT delivered less low‐dose irradiation to the heart overall. For cardiac substructures, the mean dose to the LAD was lowest with c‐VMAT (19.72 ± 5.42 Gy), which was statistically significant (*p* = 0.03). Additionally, c‐VMAT showed significantly lower V20, V30, and V40 dose volumes compared to the other two techniques (*p* < 0.05), suggesting reduced high‐dose exposure. For the left ventricle (LV), there was no significant difference in mean dose among the groups (*p* = 0.436); however, the V10 dose of c‐VMAT (18.6 ± 6.48%) was significantly lower than that of hy‐IMRT (24.67 ± 6.98%) (*p* = 0.006), indicating less low‐dose irradiation. Table 3 presents the doses to the lungs and other critical organs. The maximum spinal cord dose with c‐VMAT (2.64 ± 1.64 Gy) was significantly higher than those of hy‐IMRT (0.56 ± 0.17 Gy) and t‐VMAT (0.74 ± 0.18 Gy), with the difference being statistically significant (*p* < 0.001). There was no significant difference in mean dose to the right breast among the three plans (*p* = 0.222). For lung volumetric parameters, no significant difference was observed in the mean left lung dose (*p* = 0.38), but c‐VMAT showed significantly lower V30 and V40 values (*p* < 0.05). The mean right lung dose with c‐VMAT (1.44 ± 0.33 Gy) was significantly higher than that of the other two groups (*p* < 0.001).

**TABLE 2:** Comparison of Cardiac, LV, and LAD dosimetric parameters among the three plans (mean ± SD).

Parameters	hy‐IMRT ($\bar{x}$ ± *s*)	t‐VMAT ($\bar{x}$ ± *s*)	c‐VMAT ($\bar{x}$ ± *s*)	*P* value	*P(LSD)*		
Heart					*P*_1_	*P*_2_	*P*_3_
D_mean_ (Gy)	5.25 ± 1.06	5.03 ± 1.36	4.93 ± 1.2	0.69			
V5(%)	19.01 ± 4.76	17.56 ± 5.15	15.63 ± 3.54	0.069			
V10(%)	11.49 ± 2.97	10.26 ± 3.6	8.8 ± 3.27	0.046	0.243	0.013	0.176
V20(%)	7.07 ± 2.19	6.31 ± 2.78	5.72 ± 3.01	0.286			
V30(%)	5.19 ± 2.03	4.41 ± 2.43	3.94 ± 2.67	0.261			
V40(%)	3.64 ± 1.89	2.96 ± 2.03	2.46 ± 2.27	0.203			
LAD							
D_mean_ (Gy)	23.95 ± 4.06	21.55 ± 5.13	19.72 ± 5.42	0.03	0.128	0.009	0.243
V5(%)	74.05 ± 8.73	77.97 ± 11.76	73.08 ± 10.9	0.308			
V10(%)	63.1 ± 9.48	61.73 ± 9.76	56.32 ± 8.36	0.056			
V20(%)	50.91 ± 9.49	45.28 ± 10.24	41.56 ± 13.43	0.035	0.117	0.011	0.297
V30(%)	40.77 ± 11.33	32.17 ± 14.19	29.21 ± 16.29	0.032	0.057	0.012	0.506
V40(%)	28.21 ± 12.54	20.07 ± 17.0	16.23 ± 16.14	0.049	0.099	0.017	0.432
LV							
D_mean_ (Gy)	9.53 ± 2.6	8.71 ± 2.86	8.47 ± 2.64	0.436			
V5(%)	34.97 ± 8.97	32.3 ± 8.92	30.93 ± 7.07	0.306			
V10(%)	24.67 ± 6.98	20.77 ± 6.89	18.6 ± 6.48	0.022	0.075	0.006	0.316
V20(%)	15.3 ± 5.28	13.34 ± 6.39	12.38 ± 6.14	0.293			
V30(%)	11.12 ± 5.17	9.42 ± 5.82	8.51 ± 5.49	0.322			
V40(%)	7.85 ± 4.76	6.39 ± 4.75	5.08 ± 4.41	0.178			

*Note*: *P*_1_: hy‐IMRT versus t‐VMAT. *P*_2_: hy‐IMRT versus c‐VMAT. *P*_3_:t‐VMAT versus c‐VMAT.

**TABLE 3:** Comparison of dosimetric parameters for spinal cord, breast_R and lung for the three plans (mean ± SD).

Parameters	hy‐IMRT ($\bar{x}$ ± *s*)	t‐VMAT ($\bar{x}$ ± *s*)	c‐VMAT ($\bar{x}$ ± *s*)	*p* value	*P(LSD)*		
Spinal cord					*P*_1_	*P*_2_	*P*_3_
D_max_ (Gy)	0.56 ± 0.17	0.74 ± 0.178	2.64 ± 1.64	<0.001	0.56	<0.001	<0.001
Right Breast							
D_mean_ (Gy)	1.61 ± 1.03	2.19 ± 1.84	2.46 ± 1.72	0.222			
Lung_L							
D_mean_ (Gy)	10.02 ± 1.82	9.69 ± 1.67	9.24 ± 1.78	0.38			
V5(%)	34.43 ± 5.71	35.52 ± 5.83	33.47 ± 7.06	0.585			
V10(%)	25.74 ± 4.64	25.2 ± 4.88	23.57 ± 5.72	0.379			
V20(%)	17.83 ± 3.9	16.48 ± 3.71	15.55 ± 3.93	0.18			
V30(%)	14.02 ± 3.43	12.14 ± 2.89	10.98 ± 2.93	0.011	0.06	0.003	0.239
V40(%)	10.39 ± 2.68	8.78 ± 2.23	7.1 ± 2.2	<0.001	0.036	<0.001	0.03
Lung_R							
D_mean_ (Gy)	0.5 ± 0.06	0.78 ± 0.1	1.44 ± 0.33	<0.001	<0.001	<0.001	<0.001
V5(%)	0.14 ± 0.26	0.16 ± 0.19	0.39 ± 1.13	0.427			
V10(%)	0.001 ± 0.003	0	0.011 ± 0.034	0.158			

*Note*: *P*_1_: hy‐IMRT vs t‐VMAT. *P*_2_: hy‐IMRT vs c‐VMAT. *P*_3_:t‐VMAT vs c‐VMAT; Dmean: mean dose; Dmax: maximum dose.

### 3.3 Delivery efficiency and MUs

Regarding monitor units (MUs) and treatment time, hy‐IMRT required the least MUs (352.68 ± 30.36), followed by t‐VMAT (396.2 ± 59.41), while c‐VMAT needed the highest MUs (501.06 ± 50.97), with significant differences (*p* < 0.001). In treatment time, t‐VMAT was the shortest (50.75 ± 6.31 s) and most efficient, whereas hy‐IMRT was the longest (188.05 ± 12.5 s). As shown in Table 4.

**TABLE 4:** Parameter comparison of the machine monitor unit and treatment times for the three plans (Mean ± SD).

Parameters	hy‐IMRT ($\bar{x}$ ± *s*)	t‐VMAT ($\bar{x}$ ± *s*)	c‐VMAT ($\bar{x}$ ± *s*)	*p* value	*P(LSD)*		
Monitor unit	352.68 ± 30.36	396.2 ± 59.41	501.06 ± 50.97	<0.001	0.006	<0.001	<0.001
Treatment time (s)	188.05 ± 12.5	50.75 ± 6.31	88.25 ± 8.28	<0.001	<0.001	<0.001	<0.001

## 4 DISCUSSION

Previous studies on radiotherapy following breast‐conserving surgery for breast cancer have primarily focused on comparing conventional intensity‐modulated radiotherapy (IMRT) with volumetric modulated arc therapy (VMAT) techniques. In this study, VMAT was further classified into continuous arc VMAT (c‐VMAT) and tangential arc VMAT (t‐VMAT) based on differences in arc delivery. A comprehensive dosimetric comparison was then performed between these VMAT subtypes and hybrid IMRT (hy‐IMRT).^14^, ^15^, ^16^ The results of this study showed that all three techniques (hy‐IMRT, t‐VMAT, and c‐VMAT) were able to meet the target dose requirements, but there were differences in target dose distribution and organ‐endangering capacity among the different radiotherapy techniques, which correlates and is consistent with the findings of several previous studies.^17^, ^18^

In terms of target‐area dose distribution, for the tumor‐bed dosing plan target area (PGTV), the average dose of hy‐IMRT was significantly higher than that of t‐VMAT and c‐VMAT. c‐VMAT performed the best in terms of homogeneity and conformality, t‐VMAT was worse, and the situation was similar for PTV; from the results of the target‐area dose distribution, c‐VMAT had a better performance in terms of homogeneity and conformality. Some studies have shown^19^ that the increase in tumor control rate is positively correlated with target area conformality and homogeneity; however, excessive radiation intensity may lead to a significant increase in the probability of damage to normal tissues. Zhao H et al.^20^ compared the VMAT and IMRT techniques and found that the VMAT plan performed better in terms of target area homogeneity, and its HI and CI indexes were significantly better than those of the IMRT plan. A study by Huang Y et al.^21^ also noted that VMAT and HT plans achieved higher target coverage and better homogeneity compared to IMRT. Yu J et al.^22^ used a new small‐arc VMAT approach and compared it with conventional IMRT in terms of dosimetric parameters in prone breast cancer patients, and compared to IMRT, the small‐arc VMAT plan improved the CI and HI of the target and reduced lung tissue acceptance, similar to the results of this study. From the present study, although hy‐IMRT has the outward expansion of the conformal field,^23^ which can reduce the risk of off‐targeting due to respiratory motion, c‐VMAT is superior in terms of target area uniformity and conformality. Meanwhile, this study found that t‐VMAT also has some unique advantages. t‐VMAT two segmented arcs can achieve a similar dose distribution to hy‐IMRT while avoiding direct irradiation of the lungs and heart, which on the one hand can effectively reduce the risk of low‐dose lung‐penetrating irradiation, and on the other hand, the treatment time of t‐VMAT is significantly shortened, and it has the fastest execution efficiency among the three protocols, which can improve the machine utilization rate of the treatment center, reduce the treatment time of patients, and maybe it can be used as a priority for some special patients, so t‐VMAT can also be one of the choices of clinical protocols.^8^, ^24^ It has been shown^25^ that good target area conformity and homogeneity can improve tumor control rate and reduce normal tissue exposure. In this study, the HI and CI of c‐VMAT in PGTV and PTV were better than those of hy‐IMRT and t‐VMAT, which is in line with the findings of previous studies, which implies that c‐VMAT better concentrates the high‐dose area within the target area, while reducing the irradiation of the surrounding critical organs, and improves the efficacy of radiotherapy. This may be related to the ability of the VMAT technique to dynamically adjust the dose rate and shape of the field during treatment,^26^, ^27^ and the greater range of curvature can provide more angles and subfields to meet the dose in the target area, making the target area more conformal.

In terms of critical organ exposure, there was no significant difference between the three techniques in terms of mean cardiac dose, but c‐VMAT was lower than the other two groups in terms of cardiac V10 dose volume, mean LAD dose, and V20, V30, and V40 dose volumes, and irradiated less of the low‐dose and high‐dose regions of the heart. Radiological heart injury is one of the common complications after radiotherapy in breast cancer patients, and Jacobse JN et al.^28^ found that lowering the irradiated dose to the heart, an important endangered organ in radiotherapy, significantly reduces the risk of radiotherapy‐associated cardiac disease. In addition, patients undergoing radiotherapy who received too high a dose of irradiation to the heart also resulted in a reduction in long‐term survival. The results of this study suggest the effectiveness of c‐VMAT in reducing the amount of radiation received by the heart, providing a more favorable option for protecting cardiac function in breast cancer radiotherapy.

The V30 and V40 doses of c‐VMAT were lower in the left lung volume recipients. However, the spinal cord had a significantly higher maximum dose of c‐VMAT than the other two groups, and the mean dose of c‐VMAT in the right lung was also significantly higher than the other two groups. To analyze the reason, in this study, although the parameters of the lung, heart and healthy breast were specifically limited during the plan design, In this study focused on left breast cancer, the ‘affected lung’ refers to the left lung (ipsilateral to the tumor), and the ‘healthy lung’ refers to the right lung (contralateral to the tumor). The continuous arc would still contribute a small amount of dose to the target area with more angular dimensions of the projections of the endangered organs during rotation, and in order to satisfy the dose of the target area, the number of jumps would be supplemented from other angles, leading to an increase in the low‐dose volume of the whole body. As can also be seen in Figure 2, there is a significant increase in the irradiated volume of muscle and adipose tissue in the back of the c‐VMAT, but there is still a clear advantage in the protection of important critical organs on the affected side. In recent years, many studies have focused on the effects of radiotherapy on critical organs. Zhao J et al.^29^ showed that V5, V20, V30, Dmean of the affected lung and V5 and V10 of the healthy lung were important factors predicting the probability of radiation pneumonitis, and that lowering the dose of irradiation of the heart and the left lung, as the critical organs in post‐breast‐conserving radiotherapy for left breast cancer, could reduce the risk of radiotherapy‐associated heart disease and radiation pneumonitis. c‐VMAT has some advantages in protecting the heart and the affected lung (i.e., left lung, ipsilateral to the tumor), which may be attributed to its pull‐arc mode and dose limitation strategy for the critical organs.^30^ However, at the same time, the dose effects of c‐VMAT on the spinal cord and right lung need to be emphasized. Despite the volumetric limitation of the low‐dose region for the organs at risk in the radiotherapy plan design, the dose effects of c‐VMAT on the spinal cord and right lung need to be further optimized for some specific patients.

## 5 CONCLUSION

This study provides an important reference for the clinical development of personalized radiotherapy plans, so that clinicians can choose the most suitable radiotherapy technique for patients according to the specific conditions of tumor location, size and anatomical structure of the organs at risk, taking into account factors such as the dose distribution in the target area and the amount of the organs at risk. For example, for patients with poor cardiac function, the advantages of c‐VMAT in protecting the heart may make it the first choice; for patients with potential risks to the spinal cord or right lung, c‐VMAT should be chosen with caution, and for some patients with intolerable pain and poor tolerance, t‐VMAT may also be considered as an option. However, this study still has shortcomings, only 20 female patients after breast‐conserving surgery for left‐sided breast cancer were included, and the sample size is relatively small, which cannot fully reflect the influence of different individual characteristics on the effect of radiotherapy techniques. Future studies need to expand the sample size to cover more patients with different ages and disease characteristics to improve the generalizability and reliability of the results. When choosing radiotherapy techniques in the clinical setting, the most appropriate radiotherapy regimen can be selected based on the patient's specific conditions and the advantages of different techniques, in order to achieve the best therapeutic effect and minimize adverse effects.

## AUTHOR CONTRIBUTIONS

All authors participated in patient treatment and were involved in preparation of the manuscript. Guarantor of integrity of the entire study: Hanfei Cai. Guideline interpretation and target volume delineating: Hanfei Cai, Shilong Song. Study concepts and design: Xin Chen. Literature research and statistical analysis: Xin Chen. Plan design: Xin Chen, Xianxiang Wu. Experimental studies/data analysis: Wei Li, Nannan Qin. Manuscript preparation: Shilong Song

## CONFLICT OF INTEREST STATEMENT

The authors have no conflicts of interest to declare.

## ETHICS STATEMENT

All experimental protocols of this study were approved by the Institutional Review Board of the First Affiliated Hospital of Bengbu Medical University. All patients included into this study had given their approval to use their data for scientific research. All personal information to identify patients was removed from the image data and analyzed retrospectively.
